# Experimental investigation of influence of amide polymer on loess for subgrade

**DOI:** 10.1038/s41598-024-62503-3

**Published:** 2024-05-28

**Authors:** Jianwei Yue, Haonan Zhang, Yage Zhang, Shaopeng Xu

**Affiliations:** 1 School of Civil Engineering and Architecture, Henan University, North Section of Jinming Avenue, Longting District, Kaifeng, 475004 Henan China; 2 School of Civil Engineering, Tianjin University, Tianjin, 300072 China

**Keywords:** Amide polymer, Polymer-modified loess, Permeability, Crack resistance, Scale model, Polymers, Civil engineering

## Abstract

The effects of moisture and drying shrinkage can lead to uneven settlement, cracking, and other diseases in loess subgrade. The objective of this study was to investigate the effects of amide polymer (AP) on the permeability, mechanical properties and crack resistance of loess by orthogonal experiments. The basic properties of AP and the permeability, mechanical properties, and dry–wet variation properties of polymer-modified loess were tested, and a scale model verification and simulation analysis were conducted. In this paper, water migration in subgrade is regulated by improving the water sensitivity of loess. By reducing the variation range of subgrade water content, the stress accumulation in subgrade caused by water is weakened. The results show that the curing time and mechanical properties of AP are directly affected by the oxidant and reducing agent, and the mechanical properties of AP are compatible with the characteristics of loess. AP filled the grain gap and reduced the permeability of loess by 34.05–280.83%. The ductility of polymer-modified loess is significantly increased, and the strain of peak strength is increased by 17.21–126.36%. AP can regulate moisture change, reduce the surface tension between particles, and reduce stress concentration. The strength loss rate was reduced by 19.98–51.21% by enhancing the cracking resistance and weakening the strength loss caused by dry and wet cycling. The increase of upper layer moisture content in the scale model of polymer-modified loess subgrade is reduced by 31.38–36.11%.

## Introduction

Global warming has intensified the water cycle and expanded the frequency, range, duration, and severity of heavy rainfall events^1^. Factors such as rainfall infiltration and groundwater rise have a significant influence on the water content distribution of subgrade under balance state^2–4^. The mineral composition and micro-particles of the intact loess undergo physical and chemical reactions after being upon immersion in water. Under the alternating effects of rainfall and evaporation, the physical and mechanical properties of the loess deteriorate^5^. The original stable structure is damaged, and the strength of the soil rapidly decreases^6^. Resulting in diseases such as expansion deformation, uneven settlement, and shrinkage cracking. Consequently, controlling the cyclic wet-dry effects of water on loess and enhancing its hydraulic properties are imperative for mitigating diseases in loess subgrade.

Mechanical reinforcement and material improvement methods are often used in traditional engineering to reduce the damage of subgrade caused by moisture. Mechanical reinforcement methods usually include the replacement method and the compaction method. The replacement method involves excavating part of the loess at the base and then replacing it with high-quality fill to reduce the wet subsidence. The compaction method vibrates the foundation through repeated loading and unloading to improve its impermeability and bearing capacity. However, these methods exhibit significant disturbance to existing subgrade and are susceptible to environmental influences. Under the impact of external factors such as water erosion and vehicle loads, their reinforcement effectiveness tends to degrade rapidly. Mechanical reinforcement methods usually involve large-scale earthworks and filling. In the event of problems such as settlement or deformation of the subgrade, larger maintenance and repair work may be required, making maintenance difficult.

The material improvement method involves introducing specific materials into the soil, prompting physical or chemical reactions between the soil and these materials. This leads to changes in critical characteristics such as the aggregation state, pore distribution, and permeability channels of the soil. At present, loess is mainly improved by adding traditional improvement materials such as lime, cement, fly ash, and new improvement materials such as microorganisms and chemical curing agents. Bao et al.^7^ chose lime and fly ash as the improved materials, and studied the change rule of their shear strength and permeability coefficient. However, the improvement of permeability coefficient by lime was limited, and the final permeability coefficient was reduced by 4% to 7.7%. Zhang et al.^8^ investigated the lateral seepage effect under different types of loess-lime structures by indoor box tests and simulations. Li et al.^9^ analyzed the effect of fly ash and admixture on the disintegration of modified loess, and the test results showed that fly ash admixture had a certain inhibiting effect on the disintegration of loess. Axel et al.^10^ found that cement interacted with loess particles to form a thicker cement network, which successfully covered the pore spaces, thus improving the water absorption characteristics and mechanical properties of the loess. Lime, fly ash and cement have certain defects although they can improve the water sensitivity of loess. The brittleness of the improved loess increases, the resistance to deformation is weakened, and it is easy to crack. The initial strength of the improved soil is poor, and it has to go through a long maintenance cycle, while it is easy to swell and destroy. In recent years, with the development of material science, more new and improved materials with good properties have been applied to practical engineering.

Cheng et al.^11^ used MICP technique to reduce the resistance of loess to rainfall erosion. Jia et al.^12^ used guar gum to stabilize loess slopes and to control rainwater erosion. Sujatha et al.^13^ utilized xanthan gum as a solidifying agent to improve the geotechnical properties of the soil, resulting in a significant enhancement of impermeability. Kulkarni et al.^14^ improved the mechanical properties of soil by using colloidal nano-dioxide treatment. However, these methods tend to focus only on improving specific indicators such as soil permeability or strength, while ignoring properties such as soil shrinkage. Therefore, in order to improve the long-term performance of the subgrade such as weather resistance, it is necessary to comprehensively consider the properties of the improved subgrade soil. Including permeability, crack resistance, mechanical properties, etc.

AP is a material containing a three-dimensional network structure of hydrophilic polymer chains, which has excellent water retention properties, good elongation and elastic recovery properties. The water retention properties of amide polymer contribute to maintaining water balance in the soil, enhancing the soil's water retention capacity^15,16^, and restricting soil cracking behavior; Its excellent mechanical properties enable it to withstand large loads and stresses^17^; Its excellent elongation performance allows it to have a certain level of deformation capability under stress^18,19^, it can maintain relatively stable performance within a certain range of strain; Its elastic recovery performance enables it to quickly return to its original state after being subjected to stress^20,21^, reducing the displacement and deformation of the structure. Additionally, by adjusting its chemical structure or adding specific chemical substances^22–24^, the performance characteristics of amide polymer can be altered. This adjustability provides greater flexibility for engineering applications. Therefore, amide polymer is widely applied in various fields such as medicine^25,26^, chemical industry^27,28^, water treatment^29,30^, agriculture^31,32^, and more. Cheng et al.^33^ proposed a method to improve the tensile strength, elongation at break and compressive strength of hydrogels by using vinyl hybrid silica nanoparticles (VSNP) as crosslinking agent and in-situ polymerized polypyrrole as conductive agent. Lu et al.^34^ developed a new nanocomposite hydrogel. This nanocomposite hydrogel exhibits excellent shape memory performance and outstanding mechanical strength. Wang et al.^35^ added acrylamide (AM) for polymerization in cement-based grouting materials. A composite material with high toughness and a three-dimensional interwoven network structure was prepared. Kebede et al.^36^ discovered that the combination of amide polymers with lime, biochar, and other improvement agents can improve the stability of soil structure and enhance resistance to runoff erosion. Zhang et al.^37^ investigated the influence of different amounts of amide polymers on the water flow characteristics during the infiltration process of slightly saline water. Wang et al.^38^ mixed fly ash, anionic polyacrylamide and sandy soil to form a consolidated soil layer, which can increase the wind erosion resistance of the soil. The results indicate that amide polymers can reduce soil infiltration rate and enhance soil water retention capacity. It can be observed that amide polymers possess significant advantages on their own and exhibit good compatibility with soil particles, which improves the hydraulic properties, mechanical performance, and weather resistance of the soil.

Loess is characterized by loose structure, poor stability, poor water retention and low cohesion. It is prone to damage under water infiltration and dry and wet effects. The above study shows that AP can form cross-linked structure in the soil and increase the adhesion between soil particles, thus enhancing the stability of the soil. AP can slow down the evaporation rate of water and increase the water-holding capacity of the soil, and its own characteristics have good compatibility with the soil particles, which can improve the hydraulic properties, mechanical properties and weathering resistance of the soil. Aiming at the defects such as high brittleness, easy cracking, weak resistance to deformation, and poor weathering resistance, which exist in the current treatment methods for loess subgrade. Based on the principle of organic–inorganic material composite, the method of improving the performance of loess subgrade with AP is discussed. The performance of AP is studied and the factors affecting its performance are analyzed. The water stability and mechanical properties of polymer-modified loess are studied by orthogonal experiment. The improvement mechanism of loess subgrade is revealed from macro and micro aspects.

## Experimental materials and experimental program

### Experimental materials

#### AP

The main ingredients of the AP solution are acrylamide (AM, ≥ 99%), ammonium persulfate (APS, ≥ 98%, initiator), N, N'-methylenebisacrylamide (NMBA, ≥ 98%, crosslinker), triethanolamine (TEA, accelerator).

#### Loess

**Figure 1:**
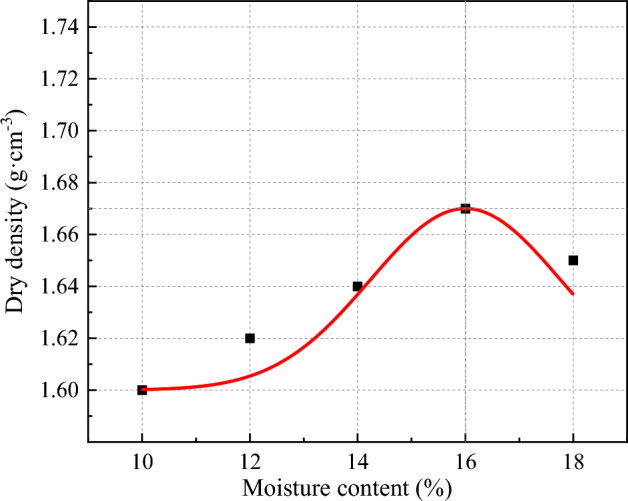
Compaction test result curve.

According to the requirements of the Standard for Geotechnical Testing Methods (GB/T50123-2019)^39^, the soil samples were subjected to percussion tests to determine the relationship between the dry density of the loess specimens and the moisture content. The results of the compaction tests are shown in Table 1 and Fig. 1. Fitting the test results, the maximum dry density of the loess was found to be 1.67 g/cm^3^ and the optimum moisture content was found to be 16%.

**Table 1:** The relationship between moisture content and dry density.

Moisture content/%	10	12	14	16	18
Dry density/g∙cm^−3^	1.60	1.62	1.64	1.67	1.65

According to the requirements of the Standard for Geotechnical Testing Methods (GB/T50123-2019), for the loess with particle size less than 5 mm used in this study, the ratio of the mass of loess particles to the same volume of pure water was measured by the specific gravity bottle method to be 2.73. Thus, the specific gravity of the loess used in the test was 2.73.

According to the requirements of the Standard for Geotechnical Testing Methods (GB/T50123-2019), the combined liquid-plastic limit method was used to measure the liquid and plastic limits of the soil. According to the specification, the liquid limit of the test sample can be obtained when the sinking depth of the falling cone is 17 mm, and the plastic limit of the test soil sample can be obtained when the sinking depth is 2 mm. The test results are shown in Table 2.

**Table 2:** Boundary moisture content test results.

Plastic limit/%	Liquid limit/%	Plasticity index	Type of soil
19.1	29.3	10.2	Silty clay

According to the requirements of the Standard for Geotechnical Testing Methods (GB/T50123-2019), cylindrical specimens with a diameter of 61.8 mm and a height of 40 mm were made for permeability testing. Variable head test was carried out using TST-55 permeameter and the coefficient of permeability of common loess was measured to be 3.37 × 10^-5^ cm/s.

**Figure 2:**
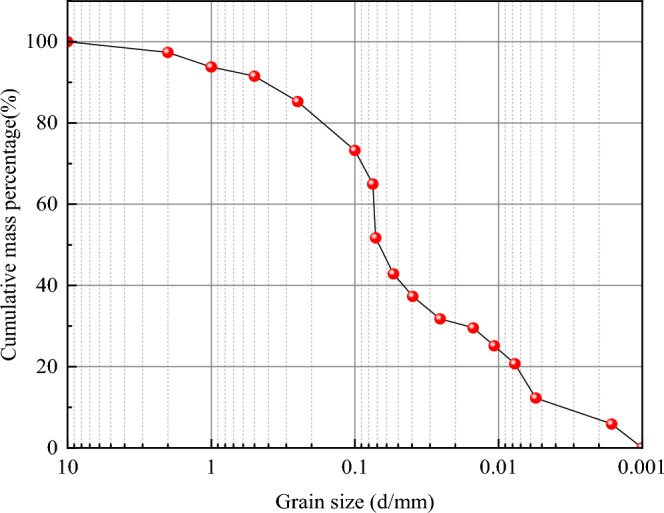
Grain size distribution.

**Figure 3:**
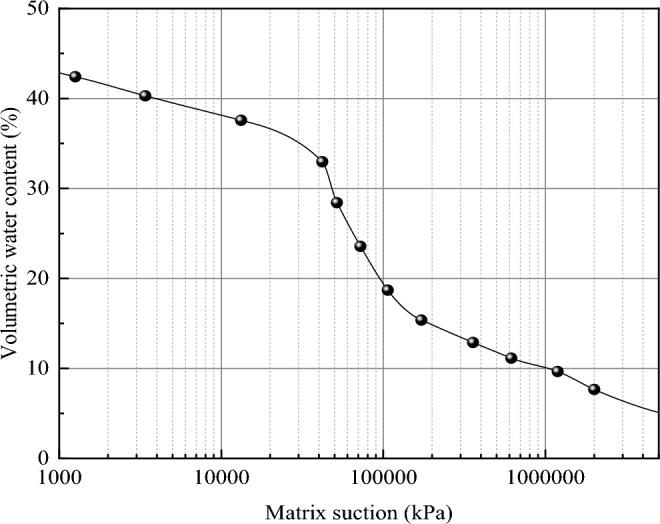
Soil–water characteristic curve.

In summary, the basic physical properties of the loess are shown in Table 3. The optimal moisture content was measured to be 16%, with a maximum dry density of 1.67 g/cm^3^. The experimental measurement of soil particle distribution is shown in Fig. 2. Using the Arya Paris model, the soil–water characteristic curve of the soil sample was drawn according to the particle size distribution and dry density of the soil sample, as shown in Fig. 3.

**Table 3:** Physical properties of loess.

Maximum dry density g/cm^3^	Specific gravity	Plastic limit	Liquid limit	Plasticity index	Saturated permeability coefficient cm/s
1.67	2.73	19.1	29.3	10.2	3.37 × 10^–5^

### Preparation of AP and polymer-modified loess

#### Configuration of AP solution

The measured acrylamide, crosslinking agent, and triethanolamine were added to deionized water, mixed thoroughly, and obtained liquid A; The measured amount of ammonium persulfate was dissolved in deionized water to obtain liquid B. Mixing liquid B into liquid A uniformly yields the AP solution. Maintaining the ammonium persulfate content at 0.01% (based on the total solution mass), the monomer AM (A), reducing agent TEA (B), and crosslinking agent NMBA (C) were chosen as the three factors for the orthogonal experimental design. The three influencing factors were arranged in combinations with three different levels, and a three-factor three-level orthogonal experiment was designed. Prepared nine formulations of AP solutions, as shown in Table 4.

**Table 4:** Orthogonal experimental design.

Sample group number	Control variables			Test piece combination number
	AM(A)/%	TEA (B)/%	NMBA (C)/%	
1	20	2	0.2	A1B1C1
2	20	3	0.6	A1B2C3
3	20	4	0.4	A1B3C2
4	25	2	0.6	A2B1C3
5	25	3	0.4	A2B2C2
6	25	4	0.2	A2B3C1
7	30	2	0.4	A3B1C2
8	30	3	0.2	A3B2C1
9	30	4	0.6	A3B3C3

#### Configuration of polymer-modified loess samples

**Figure 4:**
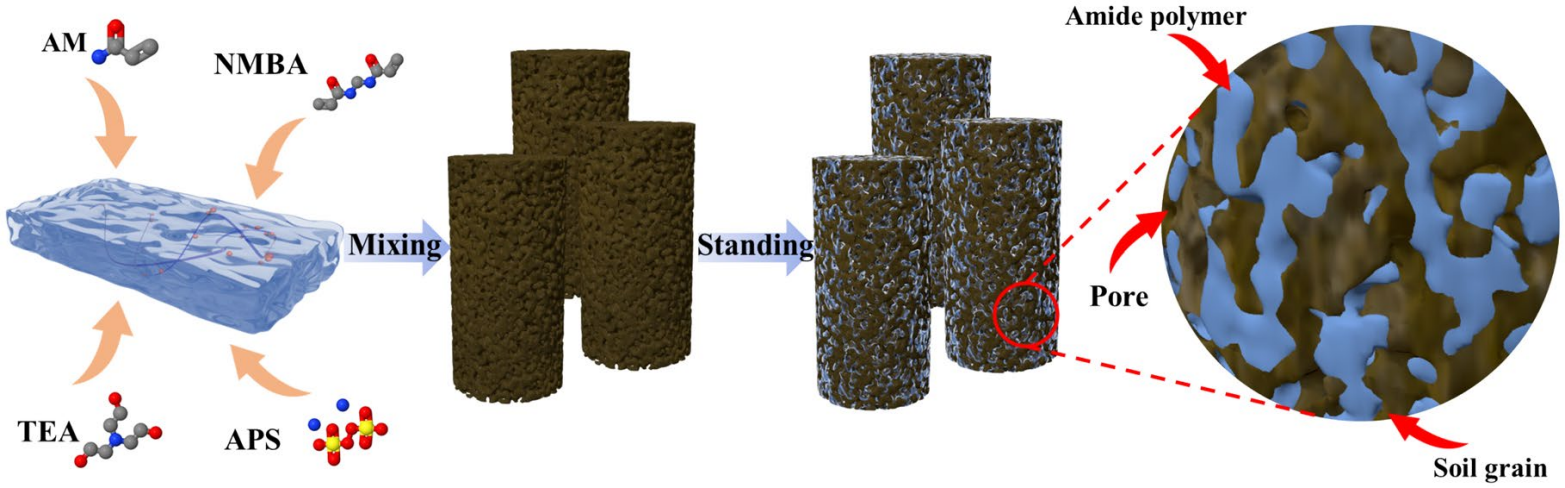
Schematic diagram of the preparation process of AP and polymer-modified loess.

Referring to the Standard for Geotechnical Testing Method (GB/T50123-2019), the process of sample preparation is illustrated in Fig. 4. Firstly, the experimental soil passed through a 2-mm standard sieve is placed in an oven and baked at 100 ℃ for 24 h to remove moisture from the soil; Secondly, the reagents are sprayed onto the soil in a wet mixing manner to prepare experimental soil with a moisture content of 16% (optimal moisture content). Before the solidification of the AP, a certain mass of soil sample is weighed according to the compaction degree of 95%, and the samples were prepared by ring knife mold and unconfined compressive strength mold. Finally, wrap the prepared specimens with plastic film and place them in a humidification chamber for curing at room temperature until the specified age (7 days).

### Preparation of AP and polymer-modified loess

#### AP performance test

##### AP water absorption and water expansion test

Water absorption: Cylindrical samples with diameter d = 61.8 mm and height h = 20 mm, with 3 samples in each group. Weigh the initial mass m_0_ of the water absorption samples. Immerse the three specimens together in 500 mL of deionized water. Retrieve the specimens every 24 h, wipe the surface to remove free water, and weigh the specimens to obtain mass m_1_. Calculate the water absorption rate (X), the result is the average of three test data, rounded to 0.01%:

1 $$X = \frac{{m_{1} - m_{0} }}{{m_{0} }} \times {\text{100\% }}$$

Water expansion: Measure the initial volume V_0_ and the volume V after 7 days of immersion of water-absorbing samples with a diameter d = 61.8 mm and height h = 20 mm using the drainage method. Calculate the water swelling rate (Y). The result is the average of three test data, rounded to 0.01%:

2 $$Y = \frac{{V_{1} - V_{0} }}{{V_{0} }} \times {\text{100\% }}$$

##### AP elongation at break test

Pour the prepared solution into polytetrafluoroethylene molds with dimensions of 10 cm × 2 cm × 1 cm, demoulded after 24 h, with 5 specimens in each group. Mark a length of 20 mm at the middle position of the tensile specimen as the original length (l_0_). Hold the ends of the specimen close to a stainless-steel ruler with an accuracy of 1 mm and uniformly stretch the sample. Record the length (l) of the marked segment at the time of sample fracture and calculate the average elongation at break (L):

3 $$L = \frac{{l - l_{0} }}{{l_{0} }} \times {\text{100\% }}$$

##### AP compressive strength test

Prepare cylindrical specimens with a diameter d = 40 mm and height h = 40 mm. Uniaxial compression experiments were performed using a universal testing machine (CMT-4204) at room temperature. The compression experiment uses the cylindrical hydrogel samples after swelling equilibrium. The cylindrical specimen was compressed to 60% strain at a rate of 2 mm/min.

#### Polymer-modified loess performance test

##### Penetration performance test of polymer-modified loess

Prepare cylindrical specimens with a diameter d = 61.8 mm and height h = 40 mm, and cure them for 7 days under standard conditions. Conduct permeability performance tests in accordance with the Standard for Geotechnical Testing Method.

##### Dry–wet cycle test of polymer-modified loess

Prepare cylindrical specimens with a diameter d = 61.8 mm and height h = 40 mm, and cure them for 7 days under standard conditions. Conduct mechanical performance tests of soil under dry–wet cycles according to the Standard for Geotechnical Testing Method. There are a total of ten sets of samples, with the first nine sets corresponding to the number of dry–wet cycles in the improved groups. The last group consists of natural soil samples subjected to n cycles (n = 0, 5, 10, 15), with four parallel samples in each group. Proceed with the preparation of specimens for dry–wet testing (optimal moisture content 16%) and adjust them to the plastic limit moisture content (19.1%). Place a permeable stone on the top surface to moisten it to the upper limit of the cycle moisture content (moisture content at equilibrium humidity + water loss rate/2). Place the samples in a humidification chamber for 24 h, then transfer them to an oven and dry them in a constant temperature and forced air circulation at 50℃ until the moisture content reaches around 16%. Seal with cling film and place it in a humidification chamber to ensure even water distribution, constituting one cycle of dry–wet cycle, repeated for a total of 15 cycles.

#### Indoor model experiment

**Figure 5:**
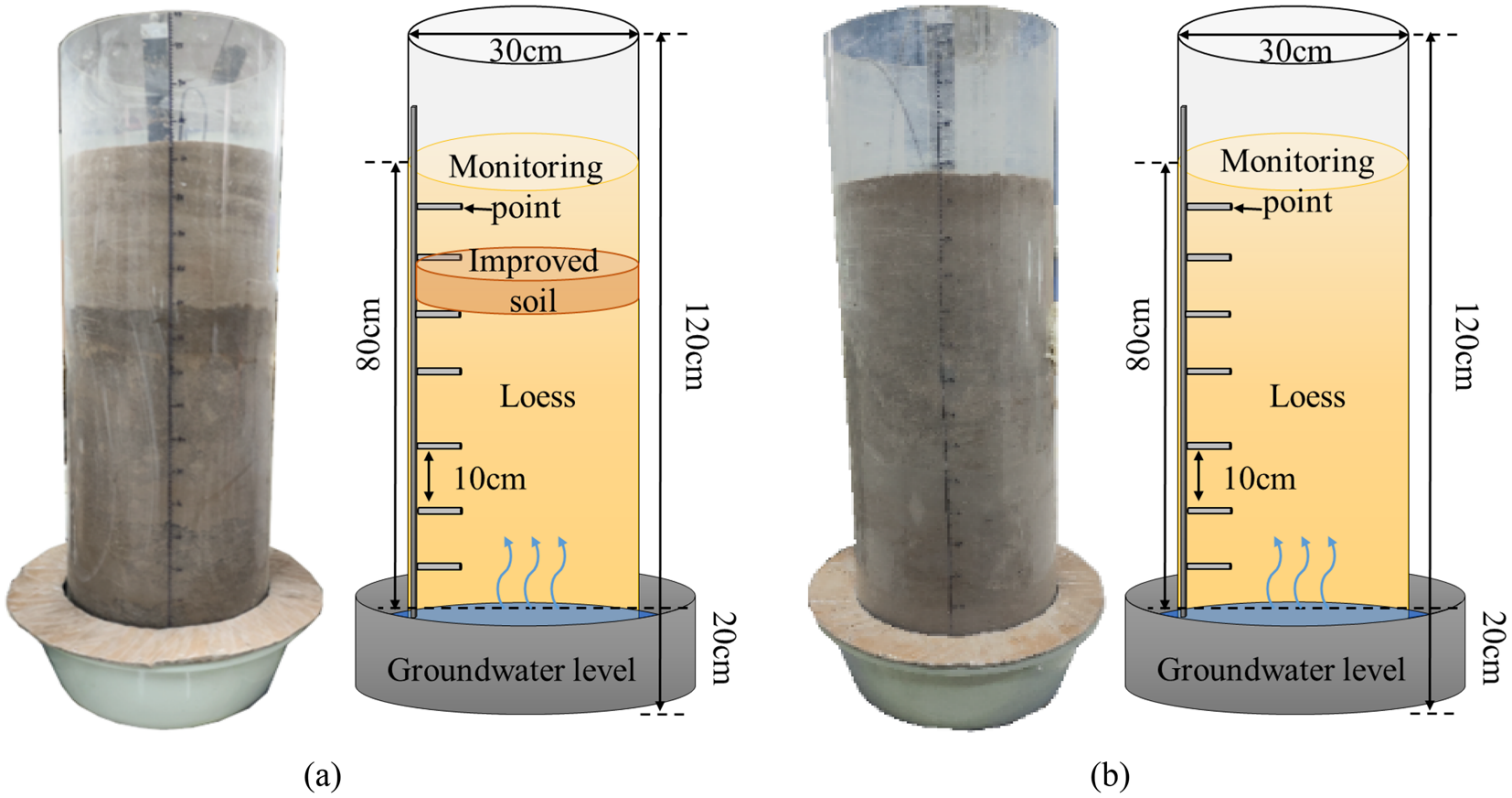
Schematic diagram of subssssgrade scaling model. (**a**) Experimental group, (**b**) Comparison group.

As shown in Fig. 5a, The plexiglass column has a net height of 100 cm, a diameter of 30 cm, and a wall thickness of 1 cm. The experimental soil samples are compacted in layers inside the acrylic column. During the compaction process, sensors for measuring soil volume, water content and temperature are installed at different depths. Soil moisture sensors for measuring soil water content are buried at depths of 10 cm, 20 cm, 30 cm, 40 cm, 50 cm, 60 cm, and 70 cm below the soil surface. A 20 cm water layer is filled to simulate the groundwater level. Using the mix proportion with the lowest permeability coefficient from Group 5, the soil layer 20 cm below the top is improved with a thickness of 5 cm. A control group is also established as shown in Fig. 5b.

## Experimental results and analysis

### Analysis of experimental results on the properties of AP

The test results for the influence of different material ratios on the performance of AP are shown in Table 5. The range analysis method was used to determine the impact patterns of each factor on the experimental indicators and the significance of each factor's impact on different indicators. The range analysis results are shown in Table 6.

**Table 5:** Results of orthogonal experiments on AP.

Sample group number	7d water absorption/%	7d water expansion/%	Elongation at break/%	Final setting time/min	Ultimate compressive strength/MPa
1	67.91	70.05	251	73	0.127
2	49.42	59.27	58	48	0.140
3	59.78	60.72	15	30	0.196
4	63.06	63.56	54	65	0.285
5	66.95	68.28	13	37	0.191
6	91.43	91.71	207	27	0.157
7	79.95	79.95	114	67	0.416
8	96.38	96.28	181	44	0.205
9	70.48	71.30	41	30	0.213

**Table 6:** Range analysis of experimental results.

Indicator factors	7d water absorption			7d water absorption			Elongation at break			Curing time			Compressive strength		
	A	B	C	A	B	C	A	B	C	A	B	C	A	B	C
$K_{1}$	177.11	210.92	255.72	190.04	213.56	258.04	324.00	419.00	639.00	151.00	205.00	144.00	0.46	0.83	0.49
$K_{2}$	221.44	212.75	221.69	223.55	223.83	223.73	274.00	252.00	69.00	129.00	129.00	97.00	0.63	0.54	0.60
$K_{3}$	246.81	221.69	182.96	247.53	223.73	194.13	336.00	263.00	153.00	141.00	87.00	143.00	0.83	0.57	0.64
$\overline{{K_{1} }}$	59.04	70.31	85.24	63.35	71.19	86.01	108.00	139.67	213.00	50.33	68.33	48.00	0.15	0.28	0.16
$\overline{{K_{2} }}$	73.81	70.92	73.90	74.52	74.61	74.58	91.33	84.00	23.00	43.00	43.00	32.33	0.21	0.18	0.20
$\overline{{K_{3} }}$	82.27	73.90	60.99	82.51	74.58	64.71	112.00	87.67	51.00	47.00	29.00	47.67	0.28	0.19	0.21
$R$	23.23	3.59	24.25	19.16	3.42	21.30	20.67	55.67	190.00	7.33	39.33	15.67	0.12	0.10	0.05

#### Analysis of water absorption and water expansion

**Figure 6:**
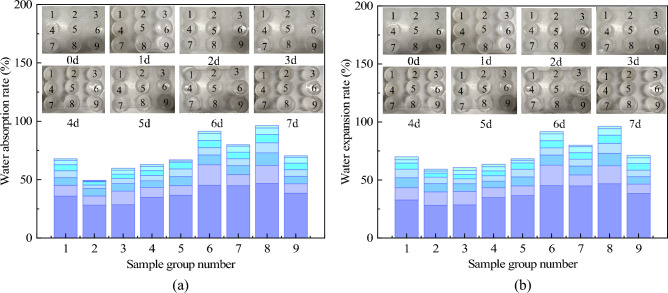
Changes in water absorption and water expansion of AP at 7d. (**a**) Change in water absorption, (**b**) Change in water expansion.

The mean values of water absorption and water expansion at each factor level, along with the range analysis results, are shown in Fig. 6 and Table 6. The water absorption rate and water swelling rate of the specimens increase with the extension of immersion time, with a faster growth rate in the early stage. When the NMBA content is 0.6%, the material is relatively stable in water. As the NMBA concentration increases, the number of crosslinking points and the crosslinking density increase, resulting in smaller pores in the network of AP, making it difficult for water to enter the network. It can be seen that within a certain range, the swelling performance of AP can be adjusted by varying the concentration of the NMBA. As shown in Table 6, the primary and secondary order of influence on water absorption and water expansion is NMBA → AM → TEA. The comparison of mean values at different levels of each factor indicates that the optimal design combination corresponding to the evaluation indicators of water absorption and water expansion is A1B2C3. That is, when the content of AM is 20%, the content of TEA is 3%, and the content of NMBA is 0.6%, the stability of AP in water is optimal.

#### Analysis of elongation at break

**Figure 7:**
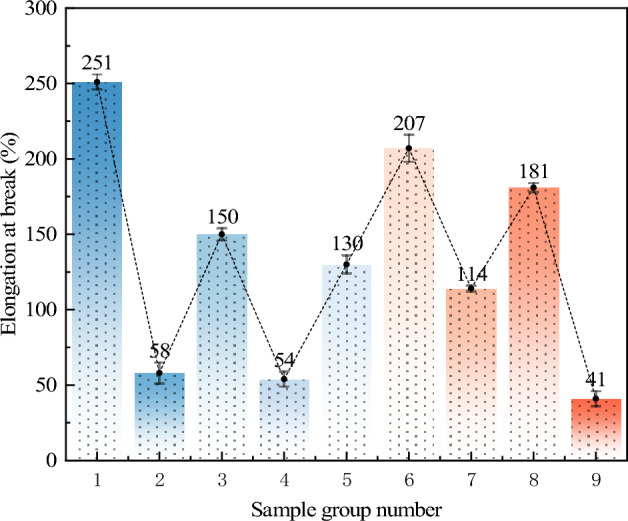
Elongation at break of AP with different ratios.

The average values of elongation at break under different levels of each factor and the results of range analysis are shown in Fig. 7 and Table 6. As the NMBA content increases, the crosslinked structure becomes denser, and the interactions between molecular chains strengthen, restricting the movement of the molecular chains. This results in the AP becoming more brittle during stretching, making it difficult to undergo plastic deformation, thereby causing a decrease in the elongation at break. According to Table 6, the primary and secondary order of influence on the elongation at break is NMBA → TEA → AM. The comparison of average values at different levels for each factor indicates that the optimal design combination corresponding to the elongation at break as the evaluation index is A1B1C1. That is, when the content of AM is 20%, the content of TEA is 2%, and the content of NMBA is 0.2%, the elongation at break of the AP is optimal.

#### Analysis of curing time

**Figure 8:**
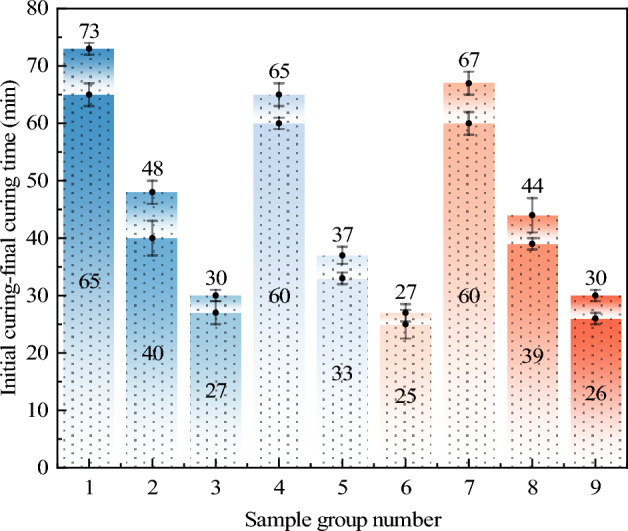
Curing time of AP in different ratios.

The mean values and range analysis results of the solidification time at different levels of each factor are shown in Fig. 8 and Table 6. Through range analysis, it is evident that the content of TEA has the most significant impact on the solidification time. With the increase in the amount of TEA, more free radicals are generated within the same time, leading to an accelerated polymerization rate and quicker solidification of AP. Adding 2% TEA based on the total mass of AP ensures a relatively extended setting time of the material, avoiding overly rapid solidification that may affect the improvement effect on subgrade soil. According to Table 6, the primary and secondary order of influence on the curing time is TEA → NMBA → AM. The comparison of average values at different levels for each factor indicates that the optimal design combination corresponding to the curing time as the evaluation index is A1B1C1. That is, when the content of AM is 20%, the content of TEA is 2%, and the content of NMBA is 0.2%, the curing time of AP is the longest.

#### Analysis of compressive strength

**Figure 9:**
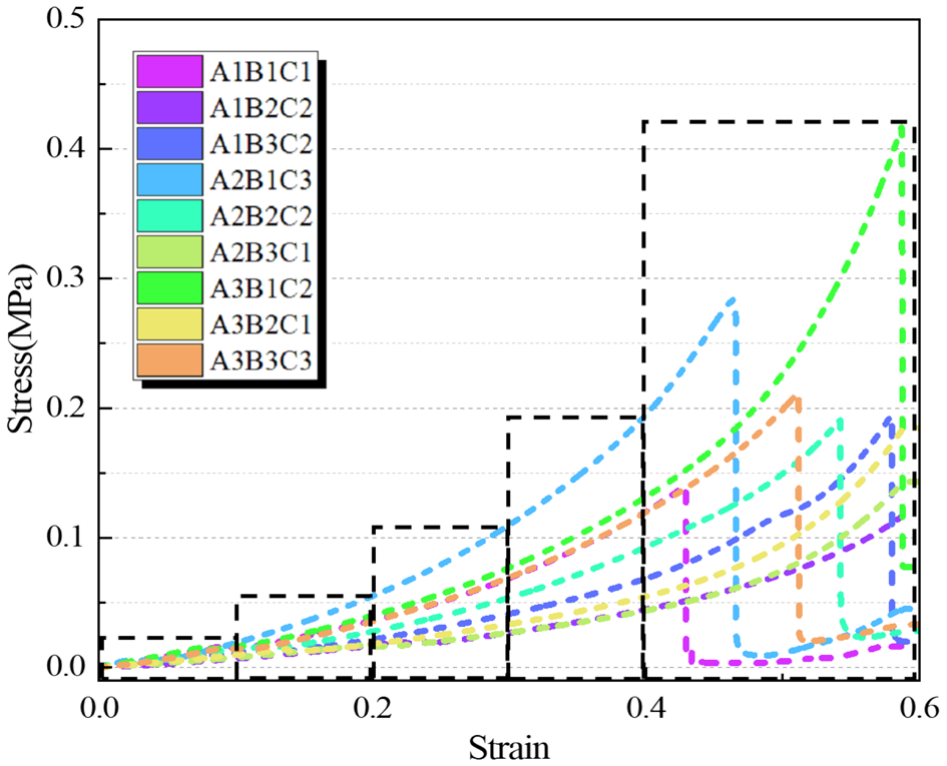
Stress–strain curves of AP with different ratios.

The stress–strain curves of AP at different factor levels and the range analysis results are shown in Fig. 9 and Table 6. The stress of AP at different ratios increases with the growth of strain until failure. According to the range analysis, the content of acrylamide monomer has the greatest impact on compressive strength. According to Table 6, the primary and secondary order of influence on the compressive strength is TEA → NMBA → AM. The increase in acrylamide monomer content leads to an increase in the length of polyacrylamide molecular chains, resulting in the formation of a more stable structure, and consequently, an increase in its strength. As shown in Fig. 9, it can be observed that the stress of amide approximately increases linearly when the strain is less than 0.1. Because within this range, molecular chains can relatively easily undergo displacement and deformation, with relatively weak interactions between the molecular chains. With the increase of strain, the molecular chain has obvious displacement, the increase of stress is increased, and gradually enters the nonlinear deformation zone. When the stress increases further, the breaking point is reached. The comparison of average values at different levels for each factor indicates that the optimal design combination corresponding to the compressive strength as the evaluation index is A3B1C2_._ That is, when the content of AM is 30%, the content of TEA is 2%, and t the content of NMBA is 0.4%, the compressive strength of the AP is optimal.

### Analysis of experimental results of polymer-modified loess

#### Penetration experiment

**Figure 10:**
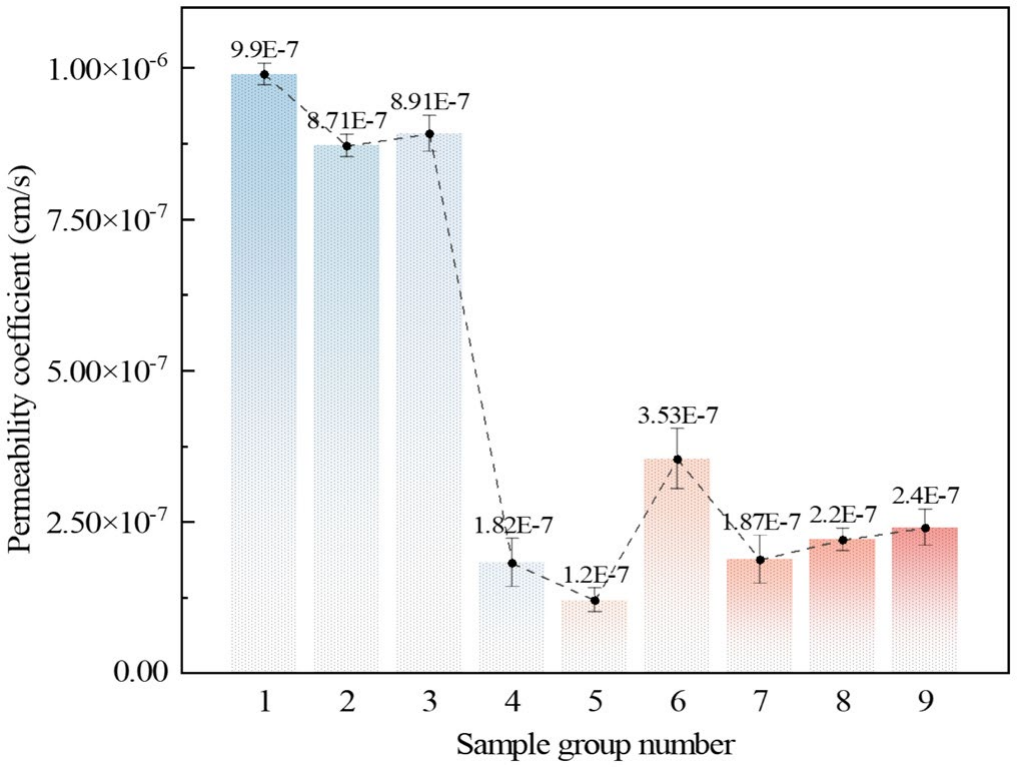
Permeability coefficient of polymer-modified loess with different ratios.

To examine the influence of AP on the permeability coefficient of loess, TST-55 permeability meters were used to conduct permeability tests on polymer-modified loess and the plain soil. As shown in Fig. 10, the permeability coefficient of the plain soil was measured to be 3.37 × 10^–5^ cm/s. After the soil was improved with the AP, the permeability coefficient ranged from a minimum of 1.20 × 10^–7^ cm/s to a maximum of 9.90 × 10^–7^ cm/s. The permeability coefficient of polymer-modified loess is 34.05%-280.83% lower than that of plain soil. Through range analysis, it is found that the order of influence on the permeability coefficient is AM → TEA → NMBA. Under the current experimental conditions, sample group 5 is considered optimal in terms of reducing the permeability coefficient of the loess.

#### Wet and dry cycle experiment

**Figure 11:**
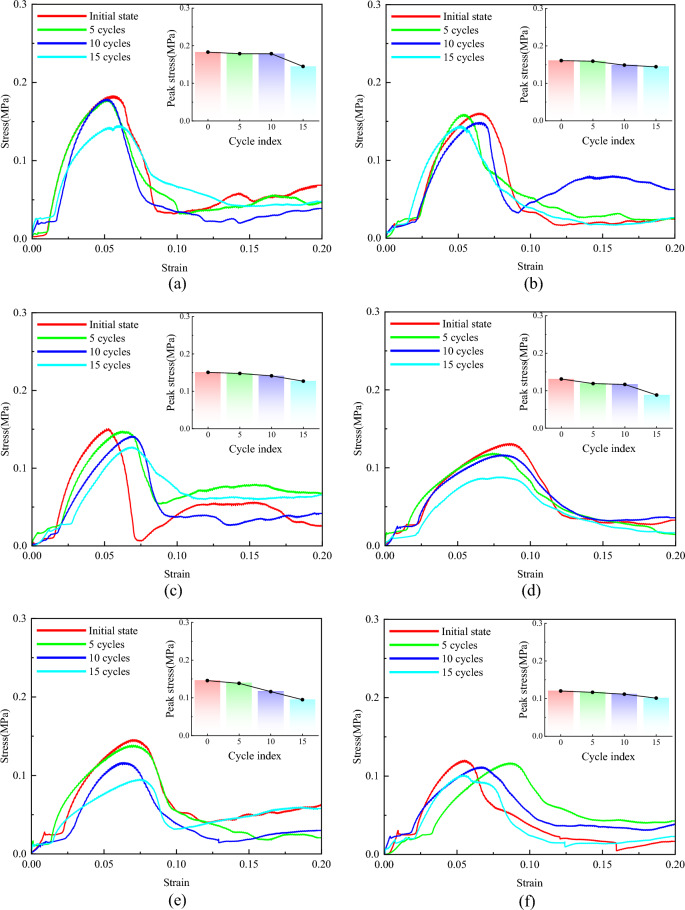

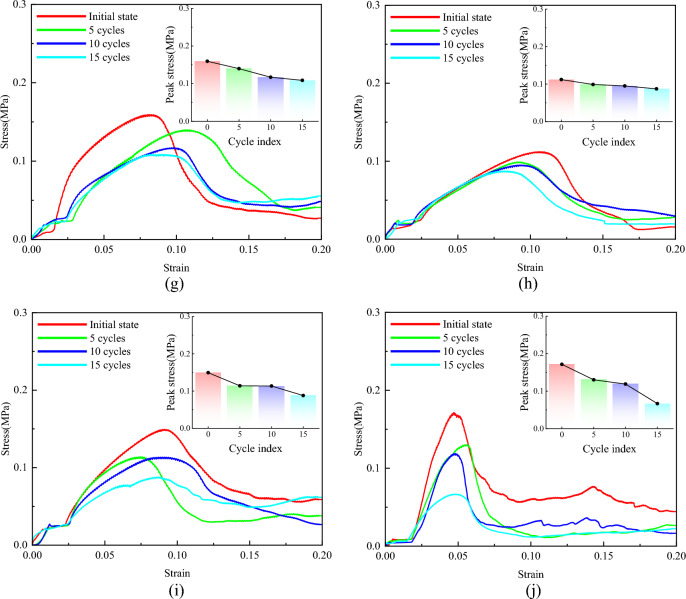
Stress–strain curves of specimens with different numbers of wet and dry cycles. (**a**) Sample group 1, (**b**) Sample group 2, (**c**) Sample group 3, (**d**) Sample group 4, (**e**) Sample group 5, (**f**) Sample group 6, (**g**) Sample group 7, (**h**) Sample group 8, (**i**) Sample group 9, (**j**) Plain soil.

As shown in Fig. 11, both plain soil and polymer-modified loess exhibit a gradual decrease in unconfined compressive strength with an increasing number of dry–wet cycles. When the dry–wet cycle is not carried out, the ductility of polymer-modified loess is improved significantly, and the strain of peak strength is increased by 17.21% ~ 126.36%. For polymer-modified loess, the occurrence of peak strength strain is obviously delayed, and the post-peak curve is gentler than that of plain soil. To further analyse the impact of dry–wet cycles on compressive strength F, we propose using the strength loss ratio (R_f_) to characterize the influence of the number of dry–wet cycles N on compressive strength:

4 $$R_{f} = \frac{{q_{u,0} - q_{u,N} }}{{q_{u,0} }} \times {\text{100\% }}$$

In the formula, q_u,0_ is the compressive strength of the specimen before any dry–wet cycles (MPa); q_u,N_ is the compressive strength of the specimen after N dry–wet cycles (MPa).

As the number of dry–wet cycles increases, there is an overall trend of increasing strength loss rate. After 15 cycles of dry–wet cycles, the peak strength of the plain soil decreased by 61.1%, with a peak strength strain of about 0.049, which remained nearly constant. The strength decay ratios for the soil samples treated with AP in groups 1–9 were 20.86%, 9.89%, 15.69%, 32.64%, 34.72%, 15.77%, 32.07%, 22.07%, and 41.12%, respectively. The variation range of peak strength strain is also very small. It can be observed that the addition of AP has a significant impact on the strength loss rate. The use of the mix proportion in sample group 2 resulted in the lowest strength loss rate, significantly improving the cyclic performance of the loess. Therefore, under the current experimental conditions, sample group 2 is considered the optimal mix proportion for enhancing the cyclic performance of loess; Sample group 8 is the optimal ratio from the point of view of improving the deformation resistance of loess.

### Analysis of indoor model experiment results

**Figure 12:**
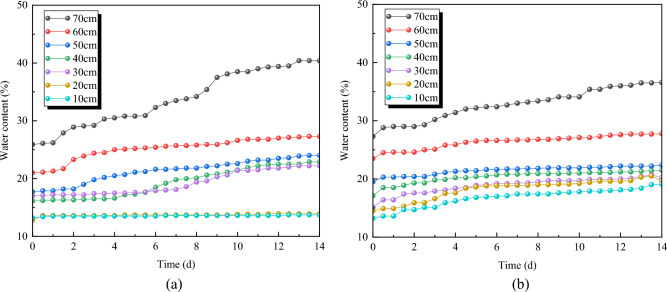
14d water content change of subgrade scaling model. (**a**) Experimental group, (**b**) Comparison group.

The change in water content for each layer over 14 days is measured and presented in Fig. 12. During the specific experimental process, it was observed that the capillary action of compacted loess subgrade is quite significant. In the early stage, the capillary water ascent speed of the subgrade soil is relatively fast. As time progresses, the ascent speed of moisture gradually slows down. Within the observed 14 days, the final increase in moisture content on the top surface of the reference group's subgrade ranged from 35.17 to 44.70%. The depth of moisture influence covered the entire model, reaching 80 cm; In the experimental group, the moisture variation range in the upper part of the improved soil layer is minimal, with a growth amplitude of 3.79 to 8.59%, and the upper part of the improved soil layer is virtually unaffected. This experimental result validates the impact of AP on the moisture distribution in the subgrade loess. The introduction of AP resulted in the restriction of soil percolation pathways, causing significant hindrance to the movement of groundwater within the improved soil layer. Moreover, the volumetric expansion of AP after water absorption contributed to a further reduction in water permeability. The combined effect of these factors significantly reduces the amplitude of moisture variation in the upper part of the improved soil layer. Therefore, the regulation of moisture distribution by AP has a pronounced effect on the subgrade. While maintaining a lower moisture content in the upper part of the improved soil layer, it effectively enhances the impermeability performance of the improved soil layer.

### Analysis of SEM results

**Figure 13:**
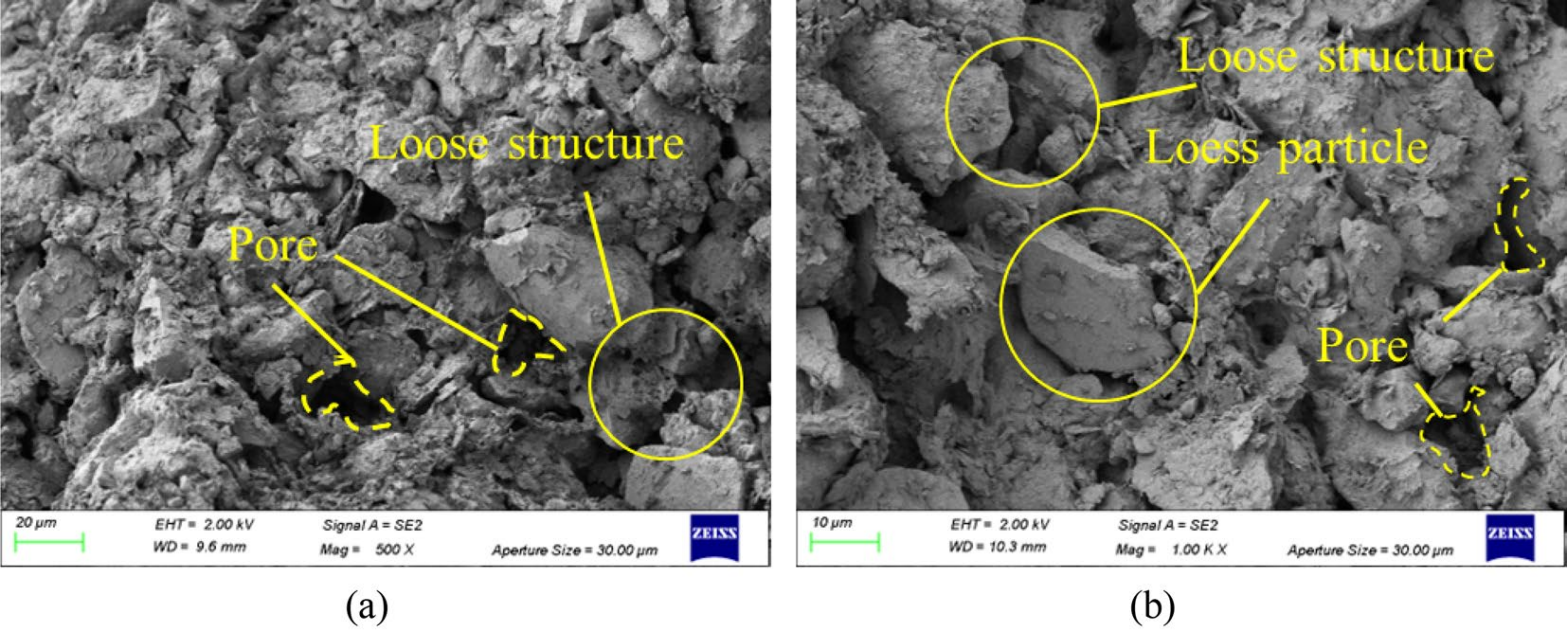
SEM images of plain loess. (**a**) 500 × magnification, (**b**) 1000 × magnification.

Figure 13 shows the SEM images of plain loess under 500 × and 1000 × magnification respectively. As can be seen from the figure, there are many voids of different sizes on the surface of ordinary loess samples, showing obvious characteristics of large pores and loose structure. A few small aggregates are distributed around the larger soil particles. The whole sample has more small particles and less cohesive polymers, and the distribution is intricate and scattered, showing a loose structure. When the soil surface is enlarged further, scattered aggregates and sheets can be observed. These aggregates and sheets are distributed on the soil surface in the form of surface contact, and some fine particles are attached to the surface of the structure through point contact. The point-to-point contact and point to surface contact between the particles are mainly, and the small and medium pores formed are more, and the corners of the soil particles are clear. In addition to the agglomeration particles of different sizes, there are some small particles in the soil. They are connected with soil particles, which together form the internal skeleton of loess.

**Figure 14:**
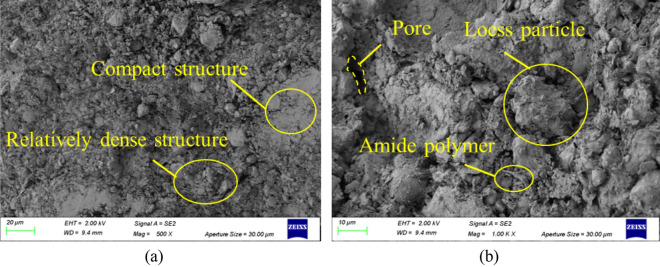
SEM images of polymer-modified loess. (**a**) 500 × magnification, (**b**) 1000 × magnification.

Figure 14 shows the SEM images of loess modified by amide polymer at 500 × and 1000 × magnification respectively. It can be found that after adding amide polymer, the porosity of improved loess is obviously reduced, and the degree of agglomeration of particles increases. The amide polymer fills the pores in the soil, making its structure tighter, and at the same time connects the particles, enhancing the integrity of the soil. The pore length and width of loess modified by amide polymer were reduced. The structure becomes more compact, and the sample appears as a large agglomeration. The particles were bonded into a whole by amide polymer, and the structural characteristics of the original soil sample were obviously changed, and the integrity of the sample was enhanced. The amide polymer filled the pores, and the number of pore channels decreased. The contact mode is transformed to surface-to-surface contact, and its own viscosity is wrapped in the outer layer of soil particles, which enhances the cementation ability of soil particles and reduces the angle of soil particles.

### Numerical simulation validation

#### Model establishment and parameters

**Figure 15:**
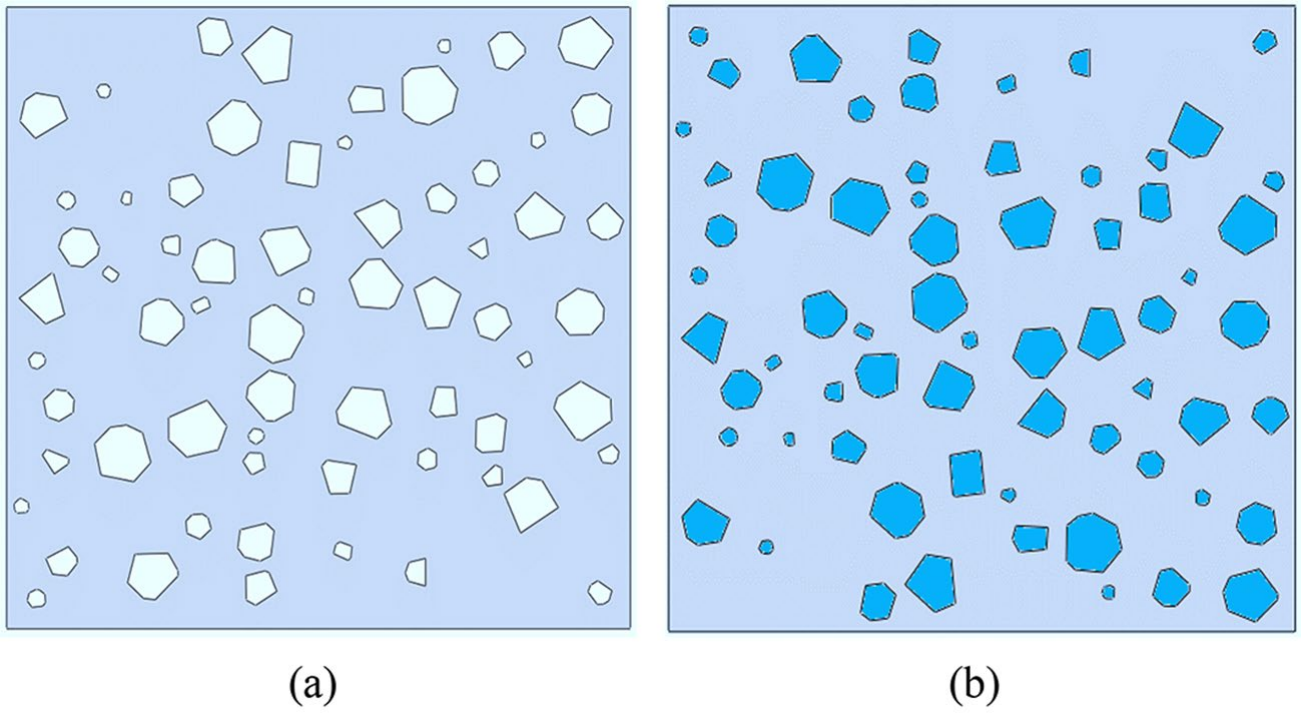
Numerical analysis model. (**a**) Plain loess, (**b**) Polymer-modified loess.

To compare the applicability performance of polymer-modified soil and plain soil in the subgrade, an analysis of the stress distribution of both materials under loading was conducted using ABAQUS finite element software. As shown in Fig. 15, the model was simplified, and a two-dimensional geometric model with a height and width of 1 mm was drawn. The soil sample model was configured as a solid entity. The model was drawn using the "CAD Random Polygon Plugin" in AutoCAD software. Porosity and its size range were input into the plugin to construct the sketch. The numerical model of polymer-modified soil consists of three parts: AP, loess, and the interface between the two. As shown in Table 7, the mesh division was set to 0.01 mm globally, with additional refinement around the boundaries and large pores; The model employed CPE4R elements with reduced integration; ABAQUS/Explicit solver module was used with a time step of 0.01 s for analysis; Loading was applied using a displacement-controlled rigid body method. The bottom two corner points were fixed in the x and y directions, and the left upper corner point was fixed in the x-direction; Periodic boundaries were set to ensure continuity of boundary stress and displacement.

**Table 7:** Model parameters and boundary conditions.

Materials	Modulus of elasticity /MPa	Poisson's ratio	Loading method	Unit type	Analysis step size /s	Boundary type
Plain loess	23	0.3	Top rigid position shift	CPE4R	0.01	Periodic boundary
Amide polymer	2.5	0.35	Top rigid position shift	CPE4R	0.01	Periodic boundary

#### Numerical simulation results analysis

**Figure 16:**
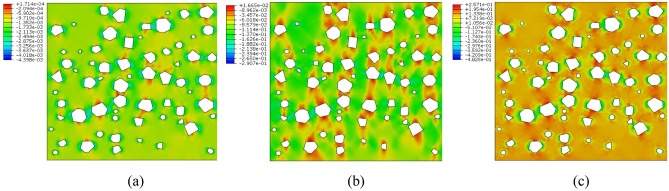
The stress cloud diagram of plain loess model. (**a**) Analysis step 1, (**b**) Analysis step 25, (**c**) Analysis step 50.

**Figure 17:**
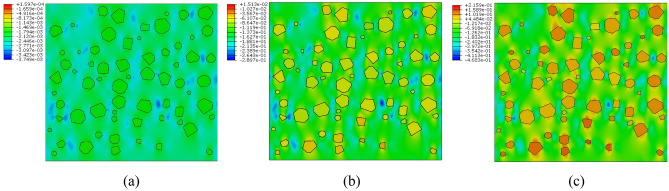
The stress cloud diagram of polymer-modified loess model. (**a**) Analysis step 1, (**b**) Analysis step 25, (**c**) Analysis step 50.

Figures 16 and 17 show the stress distribution of plain loess and polymer-modified loess respectively. As shown in Fig. 16, during the initial loading phase, the stress distribution in the soil is relatively uniform, with a small portion of stress concentrating at the tips of larger pores. As the loading progresses, the soil undergoes deformation and compression, and the presence of pores leads to a sudden change in stress around them. Around the pores, due to the hindrance from the applied load, stress concentrates on soil particles near the pores, resulting in an increase in stress values. This localized stress increase induces plastic deformation or damage to surrounding soil particles, continuously expanding the area of stress concentration. In the end, stress in different areas around the pores interconnect, allowing the transmission of stress concentration phenomena between different pores. This results in the formation of vertically coherent damage zones, leading to the failure of the specimen. As shown in Fig. 17, the presence of AP inside the pores allows it to bear a portion of the stress. In the initial stage, compared to plain soil, the stress distribution around the pores is more uniform, with no noticeable stress concentration areas. As the loading progresses, the AP undergoes compression within the pores, leading to an increasingly uneven stress distribution, with certain local areas experiencing higher stress. However, the stress around the pores does not extend significantly but is distributed uniformly in a small range around the pores. This suggests that the AP increases the contact area between soil particles, altering the path of stress transfer and reducing stress concentration.

**Figure 18:**
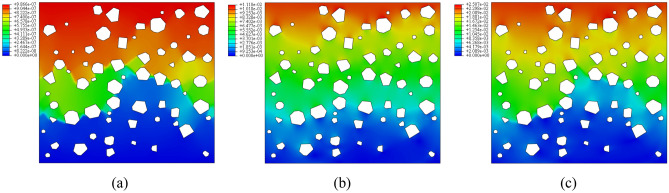
The displacement nephogram of plain loess model. (**a**) Analysis step 1, (**b**) Analysis step 25, (**c**) Analysis step 50.

**Figure 19:**
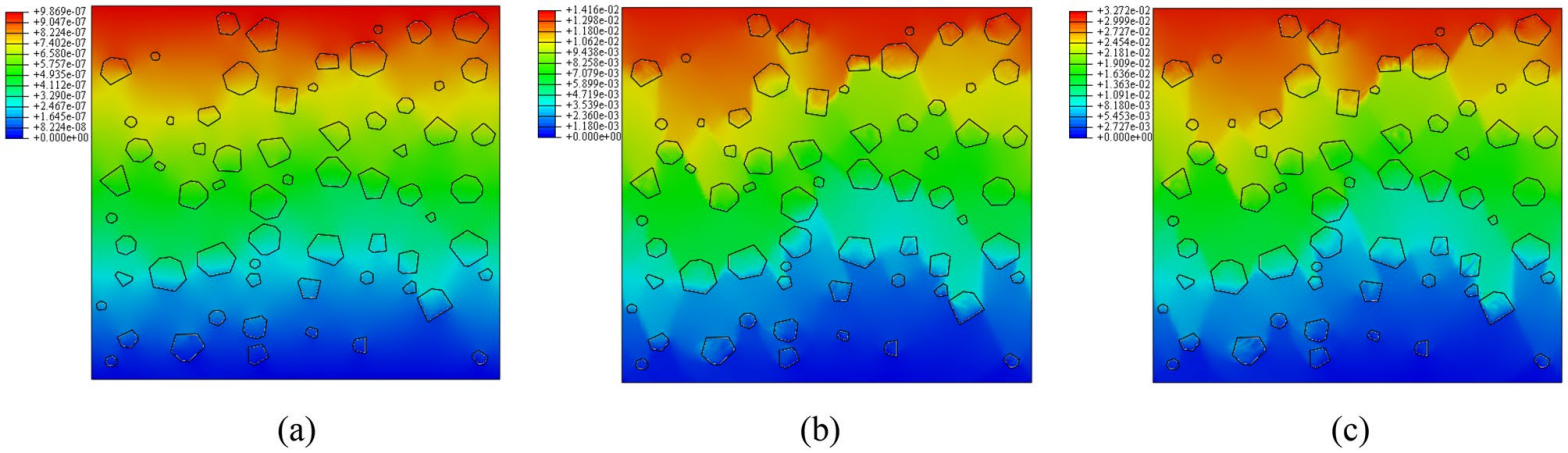
The displacement nephogram of polymer-modified loess model. (**a**) Analysis step 1, (**b**) Analysis step 25, (**c**) Analysis step 50.

**Figure 20:**
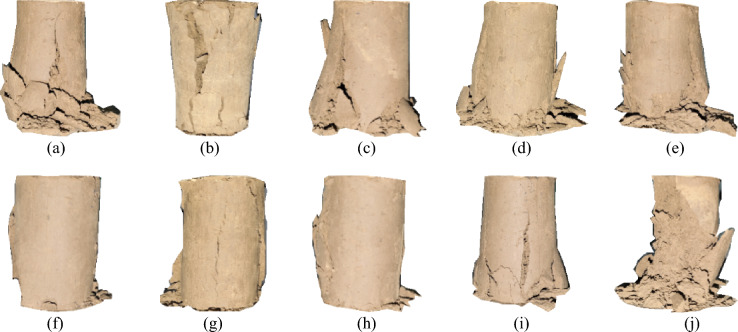
Damage patterns in plain loess and polymer-modified loess. (**a**) Sample group 1. (**b**) Sample group 2, (**c**) Sample group 3, (**d**) Sample group 4, (**e**) Sample group 5, (**f**) Sample group 6, (**g**) Sample group 7, (**h**) Sample group 8, (**i**) Sample group 9, (**j**) Plain loess.

Figures 18 and 19 depict the displacement nephogram of plain loess and polymer-modified loess. In terms of displacement over the interval from the first step to the 50th step, the displacement of plain loess increases from 9.866 × 10^–7^ mm to 2.507 × 10^–2^ mm, while the displacement of polymer-modified loess increases from 9.969 × 10^–7^ mm to 3.272 × 10^–2^ mm. This indicates that the addition of AP leads to an increase in displacement in the improved soil, delaying the occurrence of failure. Throughout the simulated loading process, the displacement increment in the polymer-modified loess is greater than that in the plain soil. This indicates that the polymer-modified loess possesses a stronger resistance to deformation. This aligns with the conclusions drawn from Figs. 11 and 20.

## Discussion and analysis of improved mechanisms

1. AP changes the confinement behavior between soil particles and effectively enhances the plastic deformation behavior of soil. The formation process of soil determines that its internal structure is loose, the pores are abundant, and the bonding degree between particles is relatively weak. These characteristics include low mechanical properties, poor plastic deformation properties, easy to be eroded by water, etc., which are the core factors affecting the instability of subgrade. AP possesses excellent mechanical properties, good elongation, and elastic recovery performance, as demonstrated in Table 6, Figs. 7, and 9. These properties are particularly suitable for modifying the performance of loess. As illustrated in Fig. 11, AP significantly influences the deformation capability of soil samples. The peak strength strain is increased by 17.21% -126.36%, which enhances the resistance of soil to the repeated influence of external environment. This characteristic is highly beneficial for roadbeds exposed to open-air environments. The AP is distributed among the soil particles (as shown in Fig. 21). At strains less than 0.1, the corresponding stress of the AP is approximately 0.03 MPa (as shown in Fig. 9), and the amide is in a linear elastic state. However, at a strain of 0.05, the stress between soil particles is significantly higher than the stress experienced by the AP. After the amide monomer undergoes polymerization initiated by APS, it will construct an AP elastic body between the yellow soil particles, binding the loess particles to form an organic–inorganic interpenetrating network structure. The amide polymer can change the pore structure of porous media and improve its deformation resistance^40^. The synergistic effect of both leads to an overall increase in the compactness of the yellow soil, significantly reducing the deficiencies in plasticity of the soil sample. The AP acts as a viscoelastic material between soil particles, enveloping them, increasing the contact area between soil particles, reducing the concentration of stress on individual particles. This helps prevent the destruction of force-transmitting particles and the continuous evolution of cracks. The presence of the viscoelastic material reduces the friction between soil particles, which is an inherent reason for the strength of the polymer sample being lower than that of the untreated soil. However, the elastic body of the polymer is also an inherent reason for improving the cyclic resistance of the AP sample. Figure 20 shows the failure morphology of specimens in each group. Through comparison and observation, it is evident that the failure morphology of specimens is significantly influenced by AP. The untreated soil suffered severe damage under pressure, with large pieces of yellow soil peeling off. With the addition of AP, the bonding interface between soil particles was strengthened, effectively suppressing the peeling phenomenon of loess within a certain range. This improved the integrity of the specimens during failure and significantly enhanced their plastic deformation capability.

**Figure 21:**
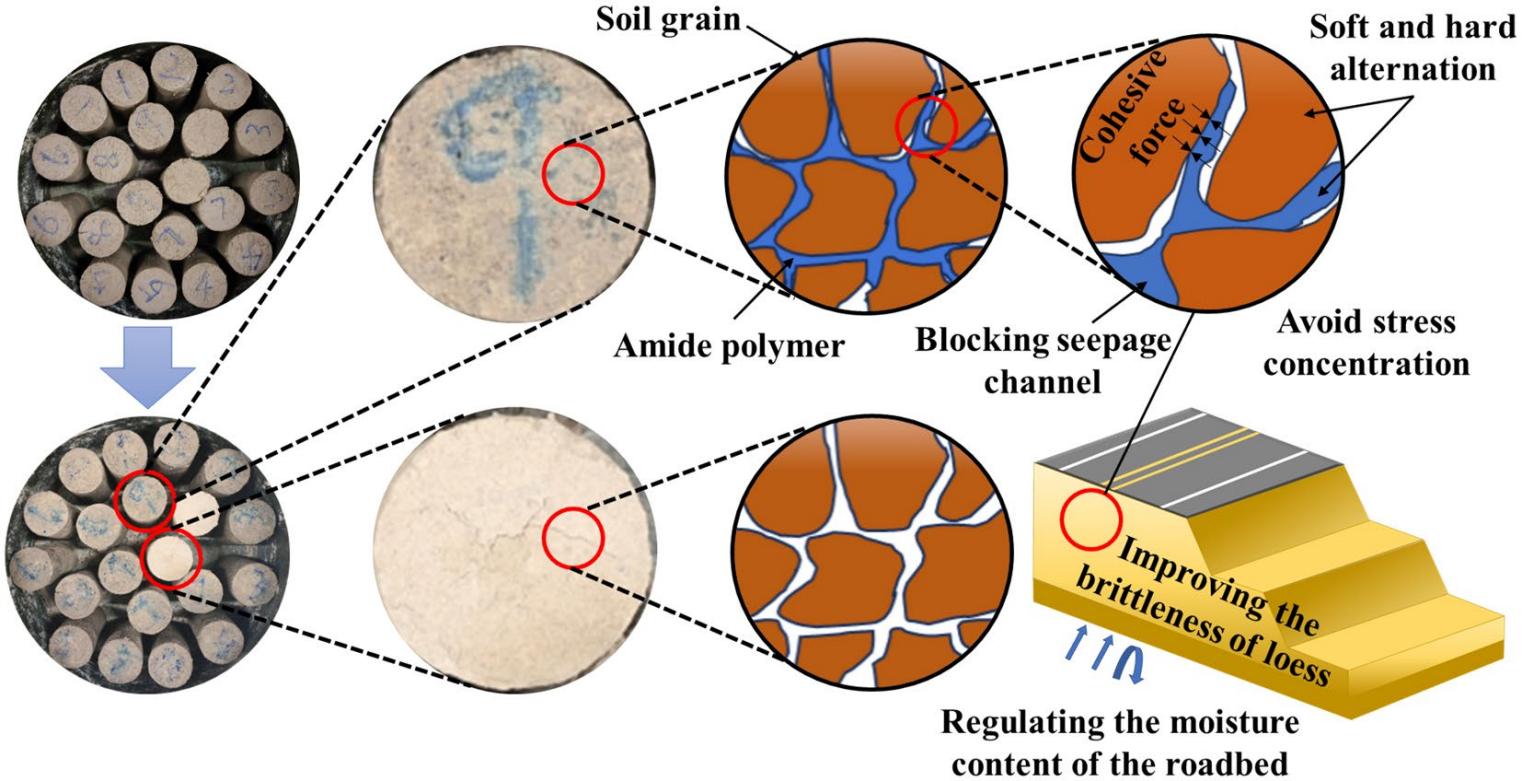
Schematic diagram of the mechanism of loess improvement by AP.
2. The AP controlled the variation range of soil moisture, reduced the surface tension between particles, decreased stress accumulation between soil particles and weakened the expansion and evolution of soil cracks. The loose soil particles in the modified soil are bonded together by AP, which enhances the compact ability of the polymer modified soil (as shown in Fig. 21), the pore structure of porous media is changed^41^, and the impermeability of porous media is greatly improved^42^. The AP filled large pores in loess, reducing the number of large pores and large seepage channels in soil. In addition, the increased curvature of the water channel results in greater resistance to water movement. This not only prevents external moisture from infiltrating but also reduces the rate of water evaporation; When the moisture between soil particles decreases significantly, soil particles absorb moisture from the interior of the AP through surface tension to replenish the moisture between soil particles. Therefore, AP can regulate the change of soil water content, reduce the evaporation rate of water in porous media^43,44^, the drying shrinkage effect is weakened (the surface tension between soil particles), and the crack resistance is improved. As shown in Fig. 22, the change in soil moisture content between soil particles determines the change in surface tension between soil particles. Fluctuations in water content led to alterations in intergranular stress and the development of intergranular cracks. The relatively stable moisture content within the soil is a prerequisite for ensuring the stability of cracks between particles. The untreated loess, after indoor static standing for 7 days, exhibited a color noticeably lighter than the improved loess. Water dispersion is serious, and there are many fine cracks on the ground surface. In contrast, the surface of the improved loess did not show any cracks. It can be observed that the judicious use of AP can alter the fundamental properties of the soil, effectively suppressing the early cracking of loess and improving its early cracking resistance.

**Figure 22:**
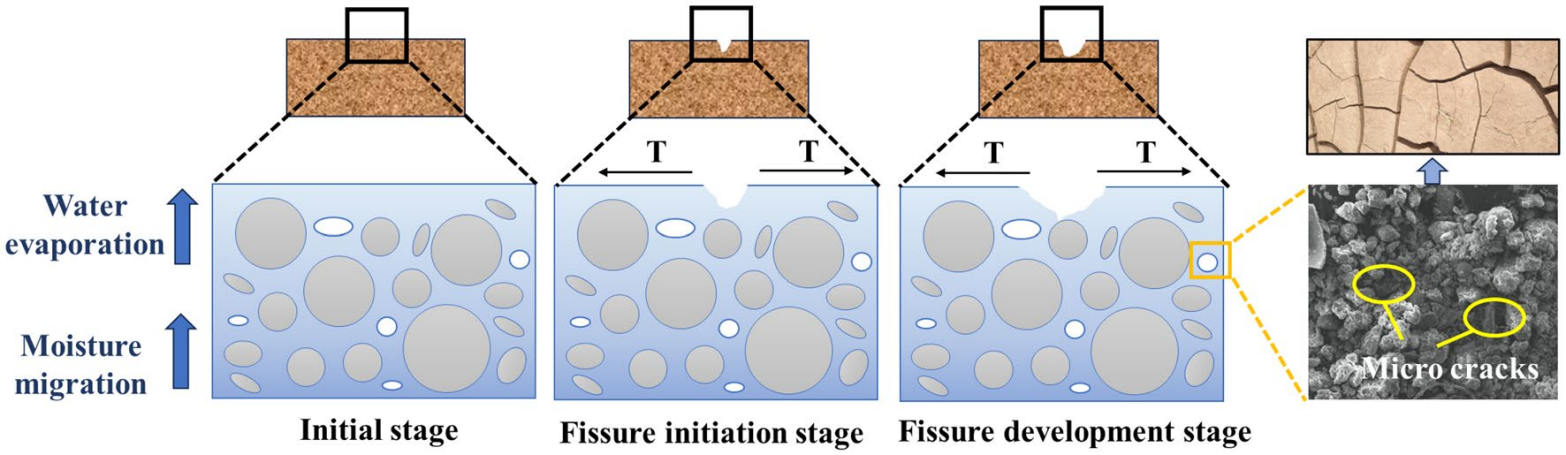
Schematic diagram of soil drying shrinkage and cracking process.
3. The AP has altered the hydraulic properties of loess and effectively reduces the height of upward movement of groundwater. After AP mixed with loess, many polymer particles were attached to the surface of loess, which wrapped the loess particles and reduced the number of small pores on the surface of loess. Additionally, AP exhibits a certain degree of expansion and compaction effect. The AP exhibits filling and water-swelling effects between particles, blocking water migration channels, thereby narrowing and convoluting the percolation paths within the soil, and extending the length of water migration routes, the permeability coefficient of the soil is reduced^45^. At the macro scale, the permeability coefficient of loess containing AP was significantly reduced (as shown in Fig. 10), the water infiltration depth was reduced (as shown in Fig. 12), and the hydraulic performance of loess was improved.

## Conclusions

The water stability and mechanical properties of loess modified by AP were studied by means of orthogonal test, permeability test, dry and wet cycle test, numerical simulation and shrinkage model test. Based on the analysis of permeability, mechanical properties and crack resistance of loess modified by AP, the following conclusions are drawn:

1. The AP has good mechanical properties and its properties can be adjusted. The strain of peak strength of AP ranges from 0.43 to 0.6, which is significantly better than that of loess. By changing the content of NMBA and AM, the water absorption, water expansion, compressive strength and elongation at break of the AP can be adjusted; By changing the content of TEA, the cross-linking reaction of the AP can be promoted and the curing time can be adjusted.
2. AP can significantly improve the anti-permeability performance of the subgrade loess. The permeability coefficient of subgrade loess after the improvement is 34.05–280.83% lower than that before the improvement. Through the study of subgrade shrinkage model, it is found that after 14 days of groundwater action, the increase of water content in the improved soil layer is 3.79–8.59%, and the increase of water content in the unimproved soil layer is 35.17–44.70%. This indicates that AP modification exhibits a significant water-blocking effect.
3. AP can effectively enhance the deformation resistance of subgrade loess. The ductility of polymer-modified loess is significantly increased, and the strain of peak strength is increased by 17.21–126.36%. Under the action of external force, the stress distribution in the structure is more dispersed than that in the untreated soil, which inhibits the displacement and deformation of loess particles, thus enhancing the stability of the subgrade loess.
4. AP can slow down the shrinkage of soil and improve its cracking resistance. The AP can control the change of soil water content and thus regulate the shrinkage of pores during water evaporation. This reduces the dry shrinkage effect of soil (surface tension between soil particles) and inhibits the initiation and expansion of early cracks in loess. After 15 cycles of wet-dry conditions, the ultimate compressive strength of untreated soil decreased by 61.10%, with noticeable surface cracks due to alternating drying shrinkage and wet expansion. In the case of improved loess, the range of the decrease in ultimate compressive strength is between 15.69% and 41.11%.

## Data Availability

The datasets used or analysed during the current study available from the corresponding author on reasonable request.
